# A Woman Suicide

**Published:** 1871-07

**Authors:** 


### A WOMAN SUICIDE.

Within a week a lady was found dead in her bed in one of the New York hotels. A note by the side of her bed stated she had no work, no money, no friends; that she had been well educated, was well connected, and had moved in good society. Nine out of ten in such cases would have borrowed or begged, or quartered themselves on indulgent friends or relations; some, — many, go on the street. No parent who reads this can certainly tell that a daughter may not arrive at such a strait in the course of a lifetime. I know a mother who, within a year, having been turned out of house and home at nightfall because her rent was not paid, walked the streets of New York a whole night, leading her little daughter by the hand, a child of eight years; and once in a while, for rest, they would stand in the doorway of some store.

The other day a wife said to me, "I have eight children; my husband was once a rich merchant; we owned our city house and country place, and rode in our own carriage; but to-day he has had but a single roll and will get no more until to-morrow."

A great deal is written and said about not being able to get work to do; the assertion is true, but the impression conveyed is false. There is abundant work for American women to do in New York city alone, to give occupation to ten thousand of them. There is work enough to do, and there are thousand of families willing to pay high prices; but there is not the skill to do it. There are multitudes of families in New York who have to wait weeks and sometimes months before their seamstresses can come to them and work for two dollars and a half a day, and fed besides; it is because they are known to be competent workers; and particular families had rather wait, and be certain that the work will be well done, and by persons of good character.

One thousand women could have employment every day in the year, at from two to six or eight dollars a day, as nurses to the sick, if they had the intelligence and tact necessary. There are thousands of families in New York, any day, who would give from twelve to twenty dollars a month, and board and lodging in elegant houses, and be thankful for the chance of getting house-girls and chambermaids, and nurses and cooks, who, fully competent, were faithful, trustworthy, and conscientious.

Five thousand of somebody's daughters, every year, go down to premature graves in New York city though the wiles of seducers, compelled to it for the want of money to house and feed and clothe them, who would not have done so, if they had possessed knowledge enough of some calling to have saved them from destitution.

This great evil must be remedied by mothers themselves, who must be educated to feel that it is a duty which they owe to their children, to society, to a common humanity, to bring up their daughters to be competent as dressmakers, as washerwomen, as chambermaids, as cooks, as nurses. Let every mother make a note of it.

